# Relationships between Dairy and Calcium Intake and Mental Health Measures of Higher Education Students in the United States: Outcomes from Moderation Analyses

**DOI:** 10.3390/nu14040775

**Published:** 2022-02-12

**Authors:** Chen Du, Pao Ying Hsiao, Mary-Jon Ludy, Robin M. Tucker

**Affiliations:** 1 Department of Food Science and Human Nutrition, Michigan State University, East Lansing, MI 48824, USA; duchen@msu.edu; 2 Department of Food and Nutrition, Indiana University of Pennsylvania, Indiana, PA 15705, USA; pyhsiao@iup.edu; 3 Department of Public and Allied Health, Bowling Green State University, Bowling Green, OH 43403, USA; mludy@bgsu.edu

**Keywords:** mental health, dairy, calcium, COVID-19 pandemic, university students

## Abstract

Background: The prevalence of mental health concerns among university students in the United States (U.S.) continues to increase, while current treatments, including medication and counseling, present shortcomings. Higher dairy and calcium intakes are associated with protective effects on mental health; however, previous studies have focused on investigating singular relationships between dairy and calcium intakes and mental health measures. A more complex exploration of these relationships is warranted to better examine whether increasing dairy and calcium intakes could serve as an intervention to improve mental health. The present study sought to further characterize the relationships between dairy and calcium intake, perceived stress, and a variety of mental health measures using linear regression and moderation analyses. Methods: The present cross-sectional study involved students studying at three large U.S. universities, and data collection occurred from April to May 2020 when students were learning remotely due to the COVID-19 pandemic. An online survey comprising validated tools was distributed among students to assess dairy and calcium intake, perceived stress, anxiety, negative and positive moods, rumination, and resilience, sleep quality and duration, dietary risk, and physical activity. Results: A total of 1233 students completed the study. Higher dairy and calcium intake was coincident with lower perceived stress and higher positive mood scores, while higher calcium intake was also coincident with lower anxiety, rumination, and higher resilience scores. Additionally, as calcium intake increased, the relationship between perceived stress and anxiety and the relationship between perceived stress and negative mood weakened. Dairy intake did not have this effect. Conclusions: Based on the results, and considering that calcium is a shortfall nutrient, universities should consider initiating programs and public health campaigns to promote dairy and calcium intake among this population.

## 1. Introduction

Mental illness is widespread in the United States (U.S.), and the recent COVID-19 pandemic appears to have further exacerbated these conditions, especially among students in higher education [1,2,3]. More than 40% of American college students report having symptoms of depression, and 80% of students report that they feel stress daily [4]. Stress, depression, and anxiety are the top factors reported by students that impact academic success [5,6]. A national college student survey reported that 63% of U.S. students stated their emotional health is worse than before the COVID-19 pandemic [7]. The prevalence of students suffering from anxiety (82%), social isolation/loneliness (68%), depression (63%), and difficulty coping with stress (60%) increased significantly during the COVID-19 pandemic compared to before the pandemic [7,8]. Globally, more than half of the students surveyed in a multinational study reported increased perceived stress during the COVID-19 pandemic [9]. However, less than half of students who have mental health concerns turned to counseling or sought help from families and friends [7]. Given the increasing prevalence of mental illness, there is a need to address mental health concerns among students in higher education.

Medication and counseling are the two most common ways to treat mental illness and reduce related symptoms [10,11]; however, there are limitations associated with both strategies. Commonly used psychiatric medications come with many side effects, including increased appetite leading to weight gain, difficulty sleeping, anxiety, restlessness, and drowsiness [12,13,14]. Additionally, previous studies indicated that less than 15% of psychiatric medication users reported high adherence to their treatment regimen [15]. Compared to medication, counseling is a more holistic way to treat and prevent mental health issues. However, when recently surveyed, only 30% of college students who said they needed counseling received treatment [7]. Common barriers to psychotherapy experienced by adults include discomfort talking about personal issues, being perceived as emotional, concerns about what others would think, cost, time constraints, transportation difficulties, or lack of childcare [16]. Given the barriers associated with the current treatments, interventions that are quick, inexpensive, and easily accessible are needed.

Ensuring adequate calcium intake may serve as a potential intervention for improving mental health, given the biological functions of calcium in the nervous system. Calcium regulates neurotransmitter synthesis and release [17], which play important roles in neuronal activation and mood regulation [18]. Additionally, calcium is required to produce serotonin, which is the precursor of melatonin [19]. Melatonin is important in regulating sleep cycles [20], and sleep plays a fundamental role in maintaining emotional health [21]. Given that dairy products provide a good or excellent source of bioavailable calcium [22], promoting dairy intake may improve certain indicators of mental health.

A small body of literature indicates that higher dairy and calcium intakes are associated with a protective effect on mental health [23,24,25]; however, these studies only investigated singular relationships between dairy and calcium intake and mental health measures. Previous studies noted associations between lower dairy calcium intake and increased anxiety [23], anger expression [26,27], depression [24,25,28], and perceived stress [26,29,30]. While these singular relationships suggest that increased dairy and calcium intake may be beneficial to mental health, the relationships between dairy and calcium intake and mental health measures are more complex than singular correlations and would benefit from further characterization.

These complex relationships stem from the fact that dairy and calcium intake may moderate the associations between stress and other mental health measures. Higher perceived stress is associated with higher levels of anxiety [9,31,32], a more negative mood [33], more frequent rumination [34,35], and lower levels of resilience [36]. Additionally, lower dairy and calcium intake are associated with higher perceived stress [26,29,37] and higher levels of other negative mental health measures, such as anxiety and negative mood [23,26,27]. Additionally, these relationships are observed for both males and females [38,39]. Given the inverse associations between dairy/calcium intake and perceived stress, anxiety, negative mood, it is plausible that individuals consuming greater amounts of dairy and calcium will report lower perceived stress and negative mental health measures.

**Figure 1:**
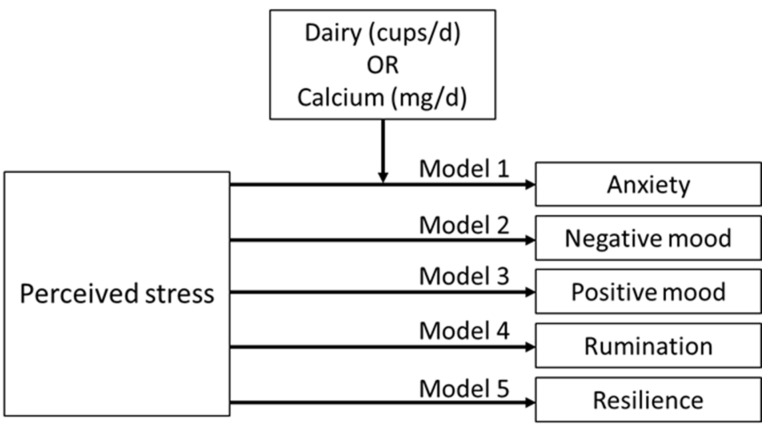
Proposed moderation models. The effects of dairy or calcium intake on the relationship between perceived stress and anxiety (moderation model 1) on the relationship between perceived stress and negative mood (moderation model 2), on the relationship between perceived stress and positive mood (moderation model 3), on the relationship between perceived stress and rumination (moderation model 4), and on the relationship between perceived stress and resilience (moderation model 5).

The purpose of this study was to characterize the relationships between dairy and calcium intake, perceived stress, and a variety of mental health measures. While dairy is a high calcium food, individuals obtain calcium from multiple food sources besides dairy, including nuts, seeds, peas, beans, fish, and calcium-fortified foods and beverages. For this reason, separate explorations of whether dairy or calcium serves as a moderator of the relationship between perceived stress and other mental health measures were undertaken. We hypothesized that: (1) both higher dairy and calcium intake predict lower levels of perceived stress, anxiety, negative, rumination, and higher levels of positive mood and resilience in higher education students in the U.S. and (2) both dairy and calcium intake moderate the relationships between perceived stress and other mental health measures, including anxiety, mood, rumination, and resilience (Figure 1).

## 2. Materials and Methods

### 2.1. Study Population and Design

The sample population was derived from a previously conducted cross-sectional study involving multiple countries and higher education institutions [40]. For the parent study, a convenience sample of undergraduate and graduate students who were at least 18 years old and studying at universities in China, Ireland, Malaysia, South Korea, Taiwan, the Netherlands, and the U.S. were recruited. From this larger sample, students studying at three large U.S. universities in Michigan, Ohio, and Pennsylvania, were included in the present study. Students outside of the U.S. were excluded from analysis for this project because the dietary questionnaires used in the present study to assess dairy and calcium intake were validated for the U.S. population. Eligible students filled out an online survey, which was delivered through Qualtrics (SAP, Provo, UT, USA) from April to May 2020 during the COVID-19 pandemic. The online survey consisted of a collection of validated tools, which are described in detail below. At the time of data collection, students at all three U.S. universities were undertaking remote instruction, and shelter-in-place orders were present. All participants provided informed consent.

### 2.2. Demographics and Anthropometrics

Eligible participants provided information regarding gender (male, female, or other), age, residency status (domestic vs. international), and class status (undergraduate vs. graduate). Body mass index (BMI) was calculated using self-reported weight and height.

### 2.3. Perceived Stress

The Perceived Stress Scale-10 (PSS-10) was employed to assess perceived stress [41]. The scale was previously validated in the university student population [42,43]. On a scale from 0 to 40, higher scores on the PSS-10 suggest more perceived stress. An example of the questions asked includes “In the last month, how often have you felt difficulties were piling up so high that you could not overcome them?” Possible responses include never (0), almost never (1), sometimes (2), fairly often (3), and very often (4).

### 2.4. Anxiety

The Generalized Anxiety Disorder Screener (GAD-7) was used to assess anxiety symptoms over the past two weeks prior to the time of study [44]. This tool is widely used in clinical settings for screening GAD [45]. An example question is “Over the last two weeks, how often have you been bothered by the following problems? Feeling nervous, anxious, or on edge”. Possible answers include not at all (0), several days (1), more than half the days (2), and nearly every day (3). GAD-7 scores range from 0 to 21. Greater scores suggest more anxiety.

### 2.5. Mood

Mood was assessed using the validated Positive Affect Negative Affect Schedule (PANAS) scale [46], which measures both positive and negative mood [47]. Positive and negative mood scores range from 10 to 50, with higher scores representing higher levels of positive mood, while lower scores indicate lower levels of negative mood. One sample question of the PANAS is “Indicate the extent you have felt this over the past week. Feeling irritable”. Possible selections include very slightly (1), a little (2), moderately (3), quite a bit (4), and extremely (5).

### 2.6. Rumination

The Repetitive Negative Thinking Questionnaire was used to examine rumination [48]. Total scores can range from 27 to 135, with higher scores being consistent with more repetitive negative thinking. The survey asks respondents how true the statements are with respect to their experience after a situation in which they felt distressed or upset. An example of a statement is “I had thoughts or images about the situation that occurred over and over again, that resulted in my feelings getting worse and worse”. Respondents answer the questions on a Likert scale ranging from 1 to 5, 1 being not true at all and 5 being very true. The questionnaire is validated for use among the general population [48].

### 2.7. Psychological Resilience

Psychological resilience was assessed using the Brief Resilience Scale (BRS) [49], which has been validated for use with a young adult population [50,51]. Resilience scores range from 1 to 5, with higher scores indicating greater resilience. A sample question is “Please respond to the following questions by marking one box per row. I tend to bounce back quickly after hard times”. Possible answers include strongly disagree (1), disagree (2), neutral (3), agree (4), and strongly agree (5).

### 2.8. Dairy and Calcium Intake

The Dietary Screener Questionnaire (DSQ) from the National Health and Nutrition Examination Survey (NHANES) 2009 to 2010 was employed to assess dairy and calcium intake from dietary sources [52]. The questionnaire assesses frequency of food consumption, including dairy and foods containing calcium, during the past month. A sample question is “During the past month, how often did you have any milk (either to drink or on cereal)? Include regular milks, chocolate or other flavored milks, lactose free milk, buttermilk. Please do not include soy milk or small amounts of milk in coffee or tea”. Frequency choices range from never to 6 or more times a day. Nutrient intake from supplements was not assessed by the survey. Intake was processed according to the DSQ Processing and Scoring protocol [53] and converted from intake frequencies to cup equivalents per day for dairy and milligrams per day for calcium. Students who self-identified as something other than male or female, e.g., transgender, genderqueer, or other, and students who chose not to disclose this information (n = 37) were excluded from analyses because the prediction equations used for estimating intake per the DSQ protocol only provided formulas for males and females.

### 2.9. Covariates

Sleep quality [24,54] and duration [54], dietary risk [55], and physical activity [56,57] were measured as covariates because they are correlated with both dairy and calcium intake and mental health measures included in the analyses.

#### 2.9.1. Sleep Quality and Duration

The Pittsburgh Sleep Quality Index (PSQI) [58] was used to evaluate subjective sleep quality. The PSQI has been validated among university students [59,60,61]. The total scores can range from 0 to 21 with higher scores indicating poorer sleep quality.

Weekend and weekday sleep duration were reported separately by participants. Average sleep duration for the entire week was calculated using the formula, (((weekday sleep duration × 5) + (weekend sleep duration × 2))/7).

#### 2.9.2. Dietary Risk

Dietary risk, for the purposes of this study, was defined as engagement in unhealthy dietary behaviors, e.g., frequent consumption of soda, fast foods, dessert, and solid fat, and infrequent consumption of fruit and vegetables. Dietary risk score was assessed using a validated simplified food frequency questionnaire, the Starting the Conversation (STC) questionnaire [62]. The STC survey measures eating frequencies of healthy (fruit, vegetables, high-quality proteins) and unhealthy foods (dessert, solid fat, chips and crackers, fast foods, and soda and sweet tea). The total scores of dietary risk range from 0 to 16 with higher scores indicating more frequent engagement in unhealthy behaviors.

#### 2.9.3. Physical Activity

The International Physical Activity Questionnaire (IPAQ) long form was used to assess physical activity [63]. The survey has been validated among young adults [64]. The total metabolic equivalents (METs) for minutes per week were calculated and reported per the IPAQ long form protocol [65].

### 2.10. Statistical Analysis

Descriptive statistics are presented as means ± standard deviations (SD) for continuous variables and as percentages (%) for categorical variables. Outliers, defined as greater or less than mean values ± 3 standard deviations, were screened and removed for all variables [66]. After outlier exclusion, all examined variables were approximately normally distributed per the normal probability plots, kurtosis, and skewness. Pearson correlation tests were conducted to determine the associations between dairy and calcium intake and mental health measures, including perceived stress, anxiety, positive and negative mood, rumination, and resilience. False discovery rate correction was used to reduce type 1 errors for multiple comparisons [67]. Additionally, linear regression was used to examine whether dairy and calcium intake predicted these mental health measures among students. Age, gender, BMI, sleep quality and duration, dietary risk, and physical activity level were adjusted in all regression models. *p* < 0.05 was used to identify statistical significance for all tests.

Moderation analyses were conducted using SPSS PROCESS Macro [68,69] to investigate whether dairy and calcium intake moderate the relationship between perceived stress and other mental health measures. A minimum of 60 students were needed to meet the power of 0.8 for each model based on 20 students per construct examined [69,70]. Model 1 in PROCESS Macro was selected for simple moderation analysis with one independent variable, one dependent variable, and one moderator. PROCESS was performed for each model by entering one independent variable (perceived stress), one moderator (dairy or calcium intake), and one dependent variable (anxiety, negative mood, positive mood, rumination, and resilience). Age, gender, BMI, sleep quality and duration, dietary risk, and physical activity level were entered as covariates for each model. Ten thousand bootstraps were performed for bias-corrected bootstrap confidence intervals. To interpret moderation analysis, the relationship between an independent and a dependent variable must be significant, and the interaction between the independent and the moderator must significantly correlate with the dependent variable in order to conclude a significant moderating relationship [69]. The DSQ data were prepared in SAS 9.4 (SAS Institute, Cary, NC, USA), and all analyses were completed using IBM SPSS version 27 (IBM Corporation, Armonk, NY, USA).

## 3. Results

A total of 1233 students completed the survey (Table 1). The majority of the students were female, undergraduates, and domestic students who were defined as students studying in their country of origin. The average BMI for both male and female students was classified as overweight.

### 3.1. Correlations

Dairy intake was negatively correlated with perceived stress and positively correlated with positive mood (Table 2) but did not correlate with anxiety, negative mood, rumination, or resilience. For calcium, intake negatively correlated with perceived stress and anxiety and positively correlated with positive mood and resilience; there were no associations with negative mood or rumination.

### 3.2. Linear Regression

Perceived stress: Linear regression analyses showed that dairy intake predicted perceived stress scores (B = −0.717, *p* = 0.031), and for every one cup equivalent increase in dairy intake, perceived stress scores decreased by 0.7 points (3%). Further exploratory analyses were undertaken to examine the effects of increasing calcium intake by 500 mg, as most studies investigating the effects of calcium on health outcomes, such as hypertension, supplement calcium intake by 500–1500 mg/d [71,72]. Importantly, increasing calcium intake by 500 mg/d does not increase the risk of developing kidney stones, even among people who have an adequate calcium intake [73,74]. Given the average intake of the study population was far below the upper limit of calcium intake (2500 mg/d) [75], a 500 mg calcium intake increase was determined to be safe and reasonable for examination. The analysis revealed that for every 500 mg increase in calcium intake, perceived stress scores decreased by 2.5 points (12%) (B = −0.005, *p* < 0.001).

Anxiety: Dairy (B = −0.121, *p* = 0.662) intake did not predict anxiety scores. However, calcium intake did predict anxiety scores (B = −0.002, *p* = 0.01). For every 500 mg increase in calcium intake, anxiety scores were reduced by 1 point (11%).

Negative mood: Dairy (B = −0.006, *p* = 0.987) and calcium intake (B = −0.002, *p* = 0.066) did not predict negative mood scores.

Positive mood: Dairy (B = 0.900, *p* = 0.016) and calcium (B = 0.005, *p* < 0.001) intake predicted positive mood scores. For each cup increase in dairy intake, the positive mood score increased by less than 1 point, i.e., 0.9 (3%), and for every 500 mg increase in calcium intake, the positive mood score increased by 2.5 points (9%).

Rumination: Dairy intake (B = 0.191, *p* = 0.864) did not predict rumination scores. However, calcium intake predicted rumination scores (B = −0.007, *p* = 0.044), and for each 500 mg increase in calcium intake, rumination scores decreased by 3.5 points (4%).

Resilience: Dairy (B = 0.017, *p* = 0.642) intake did not predict resilience scores, but calcium intake did (B = 0.0003, *p* = 0.003). For every 500 mg increase in calcium intake, the resilience score increased by 0.15 (5%).

### 3.3. Moderation Analyses

Findings from the moderation analyses indicate that perceived stress was positively associated with anxiety (Table 3); however, dairy intake was not associated with anxiety, and the interaction between perceived stress and dairy intake was also not associated with anxiety. This finding indicates that dairy intake did not moderate the relationship between perceived stress and anxiety. In contrast, calcium intake was associated with anxiety, as was the interaction between perceived stress and calcium intake and anxiety (Table 4). These results suggest that calcium intake moderated the relationship between perceived stress and anxiety. Further, we also conducted conditional effect examinations, which analyzed the relationships between perceived stress and anxiety at the 16th, 50th, and 84th percentiles of calcium intake of the sample population These percentiles were used based on the median value of calcium intake (50th percentile; 909 mg/d), one Z-score below the median (16th percentile; 806 mg/d), and one Z-score above (84th percentile; 1144 mg/d) to best reflect the calcium intake distribution of the study sample [69]. The strength of the relationship between perceived stress and anxiety decreased as the calcium intake level increased from 806 mg/d to 909 mg/d and from 909 mg/d to 1144 mg/d. To summarize, the relationship between perceived stress and anxiety weakened as calcium intake increased.

Perceived stress was positively associated with negative mood, but dairy intake and the interaction between perceived stress and dairy intake were not (Table 5). This result indicates that dairy intake did not moderate the relationship between perceived stress and negative mood. However, calcium intake and the interaction between perceived stress and calcium intake were associated with negative mood (Table 6), indicating that calcium intake moderated the relationship. Further, the relationship between perceived stress and negative mood was once again examined at the 16th, 50th, and 84th percentile calcium intake level of students [68]. The strength of the relationship between perceived stress and negative mood decreased as the calcium intake level increased from 806 mg/d to 909 mg/d and from 909 mg/d to 1144 mg/d. Taken together, as calcium intake increased, the relationship between perceived stress and negative mood weakened.

Perceived stress was negatively associated with positive mood, but dairy and calcium intake were not (Table 7 and Table 8). Further, the interactions between perceived stress and dairy intake and between perceived stress and calcium intake were not associated with positive mood. Thus, dairy and calcium intake did not moderate the relationship between perceived stress and positive mood.

Perceived stress was positively associated with rumination; however, dairy and calcium intake were not (Table 9 and Table 10). In addition, the interactions between perceived stress and dairy intake and between perceived stress and calcium intake were not associated with rumination. Based on these results, neither dairy nor calcium intake moderated the relationship between perceived stress and rumination.

Perceived stress was negatively associated with resilience, yet dairy and calcium intake were not (Table 11 and Table 12). The interactions between perceived stress and dairy intake and between perceived stress and calcium intake were also not associated with resilience. These results indicate that neither dairy nor calcium intake moderated the relationship between perceived stress and resilience.

## 4. Discussion

The study sought to characterize the relationships between dairy and calcium intake and mental health measures that included perceived stress, anxiety, negative and positive mood, rumination, and resilience. Additionally, the study explored whether dairy and calcium intake moderate the relationship between perceived stress and other mental health measures. The results demonstrate that dairy and calcium intake predicted perceived stress and positive mood scores while calcium also predicted anxiety, rumination, and resilience scores for higher education students in the U.S. The moderation analyses indicated that calcium, but not dairy intake, moderated the relationship between perceived stress and anxiety and the relationship between perceived stress and negative mood. Further, when calcium intake increased, the relationship between perceived stress and anxiety weakened as did the relationship between perceived stress and negative mood. While effects were modest, the cumulative effects of small changes could be quite substantive. Taken as a whole, these data suggest that increasing dairy and calcium intake among U.S. higher education students may serve as an intervention to reduce stress, anxiety, and rumination to improve mental health. However, these conclusions need to be empirically tested due to the cross-sectional nature of the present study.

While a link between dairy and calcium intake and mental health might not be obvious at first, there is at least one biological mechanism that explains how dairy and calcium intake could lead to improvements in mental health and perceived stress. Low serotonin levels are associated with higher anxiety [76] and perceived stress [77,78]. Serotonin production requires both tryptophan and calcium, both of which are abundant and biologically available in dairy [19,79]. Further, serotonin is the precursor for melatonin, which helps regulate the sleep–wake cycle [80]. Good quality of sleep as well as an adequate amount of sleep is essential for maintaining mental health [81,82]. Thus, there is a plausible mechanism that explains how adequate dairy and calcium consumption supports mental health.

In some cases, it was observed that calcium had positive effects on mental health measures but not dairy. This lack of findings for dairy might be explained by the fact that we were unable to distinguish high-fat dairy from low-fat dairy due to the dietary assessment tool used. For example, the present study observed higher calcium intake predicted lower anxiety, but not higher dairy intake. The literature regarding the relationships between dairy intake and anxiety is mixed, with some studies noting an inverse relationship between total dairy intake and anxiety [83,84] and others reporting greater consumption of high-fat dairy was associated with more severe anxiety symptoms or that only low-fat dairy was associated with better mental health [26,85,86]. The present study did not quantify high-fat versus low-fat dairy intake, which could contribute to the null results.

### 4.1. Correlation/Regression Analyses Discussion

As hypothesized, higher dairy and calcium intake predicted lower perceived stress and a more positive mood. These findings are similar to what was previously reported; lower dairy and calcium intakes were associated with higher perceived stress [26,29]. Additionally, a randomized crossover study on Australian adults noted that diets supplemented with 3 to 4 servings of dairy foods per day improved mood and decreased anger [87]. As for the relationship between calcium intake and mood, among adults with mood disorders, higher calcium intake was associated with better psychiatric functioning [88]. As explained above, higher dairy and calcium intake provides ingredients essential for serotonin synthesis, which promotes better regulation of sleep and mental health [19,79,80]. Thus, associations between higher dairy and calcium intakes and lower perceived stress and better mood are consistent with both previous literature and our understanding of physiology.

That higher calcium intake, but not dairy intake, predicted lower anxiety scores was surprising. Calcium intake predicting anxiety aligns with what was previously reported in two recent cross-sectional studies on university students in Jordan [23,24]. Regarding the relationships between dairy intake and anxiety, previous research noted inconsistent results and was conducted in a wide variety of populations [26,83,84,85,86]. As discussed above, not distinguishing between high-fat versus low-fat dairy intake may have contributed to the null result. Given the inconsistent results regarding the relationships between dairy intake and anxiety and the heterogeneity of the study populations of previous studies, it is too early to make definitive dietary recommendations regarding dairy intake and its effects on anxiety.

We are not aware of any previous study that has explored the relationships between calcium and dairy intake and rumination. Results from the present study indicate that higher calcium intake predicted lower rumination, but higher dairy intake did not. The lack of association between dairy intake and rumination could be due to the inability to differentiate between high- and low-fat dairy consumption, as discussed previously. An interventional study exploring the effects of calcium intake on rumination and anxiety is warranted.

Higher calcium intake, but not dairy, also predicted higher resilience scores. Resilience is defined as the ability to cope with a stressful situation or crisis and to return to the precrisis mental state quickly [89]. Higher resilience is associated with lower perceived stress [9,90], reduced anxiety, and higher positive mood scores [91]. Given that resilience was associated only with calcium intake, this might explain why dairy intake was not associated with resilience scores. The possibility of increased calcium intake as an intervention to improve resilience is worthy of future study.

### 4.2. Moderation Analyses Discussion

As hypothesized, calcium intake moderated the relationship between perceived stress and anxiety and the relationship between perceived stress and negative mood. These findings agree with previous studies where inverse associations were reported between perceived stress and calcium intake [26,29,92] and negative mood [26,27]. Results from the present study also indicate that as calcium intake increased, the association between perceived stress and anxiety weakened. Biologically, calcium serves as a neurotransmitter that plays an important role in emotional regulation [93]. Extracellular calcium disturbance can lead to mood disorders and increased stress [93], and adequate consumption of calcium is a key component in the maintenance of cellular calcium balance [94]. Given the role that calcium plays in emotional regulation and the results of the present study, increasing calcium intake could be effective in improving mental health of students by reducing stress-induced anxiety.

Contrary to our hypothesis, dairy intake did not moderate the relationships between perceived stress and other mental health measures including anxiety and both negative and positive mood. Recent publications indicate inconsistent results regarding the relationships between dairy intake and anxiety [26,83,84,85,86], and some studies point out that only higher intake of low-fat dairy is associated with lower anxiety symptoms [26,85,86]. The present study did not quantify dairy based on fat content, which could explain why dairy intake did not moderate the relationship between perceived stress and anxiety. In terms of negative mood, previous studies focused on investigating the association between dairy intake and one specific mood state, such as anger expression [26,27], tension [95], depression [95], and confusion [87], not the overall negative mood expression. Therefore, it is difficult to compare our study results to the body of literature as the outcome measures are different. When examining the relationship between dairy intake and positive mood, several previous studies investigated the effects of consuming raw or fermented raw milk [96,97] and observed that raw milk consumption was associated with a positive mood change. One cross-sectional study examining the relationship between Mediterranean food intake and positive and negative mood noted that dairy intake, including both high-fat and low-fat dairy, was positively associated with positive mood only in females [98]. Compared to previous studies, the focus of the present study was not on specific types of dairy or gender-specific outcomes, which may explain the null effect of dairy intake on the relationship between perceived stress and positive mood. However, the present study adjusted gender as a covariate in the regression and moderation analyses. To summarize, our results do not support increasing dairy intake to improve mood.

### 4.3. Public Health Message

These results suggest that increased dairy and calcium intake are associated with better mental health measures, particularly for individuals who are not consuming enough dairy and calcium. The recommended calcium intake for young adults is between 1000 mg to 1300 mg based on age [99]. In general, university students do not meet dairy and calcium intake recommendations, and consumption of these nutrients by students has trended downwards over time [99,100], while mental health issues have trended upwards [1,2]. The moderation analyses of the present study suggested that even small increases in intake, as evidenced by an increase from approximately 800 mg/d of calcium to 900 mg/d, appear to have a beneficial effect on mental health. Given the available data, public health campaigns targeted to young adults that emphasize the benefits of dairy and calcium consumption on mental health should be tested.

When considering an intervention designed to increase dairy and calcium intake in this population, several factors should be considered. Previous research noted that adolescents experienced decreased calcium consumption when transitioning to young adulthood due to several factors, including: mealtime dairy availability, health/nutrition attitudes towards dairy, taste preferences, weight control behaviors, and peer pressure [101]. These factors should be considered when developing an intervention to increase intake in this population. Additionally, when promoting dairy- and calcium-rich food consumption, appropriate educational channels for young adults should be considered, such as social media [102]. Overall, a successful program that promotes dairy and calcium consumption will need to consider factors that influence university students’ dairy and calcium intake and their preferred communication platform.

### 4.4. Strengths and Limitations

The present study possesses multiple strengths. First, the study population consisted of students from three large universities in the U.S. and included undergraduate, graduate, domestic, and international students, which makes the study results more generalizable to U.S. higher education students as a whole. Second, the large sample size of the study allowed for adequate power to conduct the analyses. Third, moderation analyses provided the opportunity to examine the complex relationships between dairy and calcium intake and mental health measures. Fourth, the regression and moderation analyses adjusted multiple lifestyle covariates, including dietary risk, sleep, and physical activity. Finally, the study used the DSQ from the NHANES 2009 to 2010, which was well-validated among U.S. adults, to quantify dairy and calcium intake.

Several limitations need to be considered when interpreting the results. First, the cross-sectional nature of the study can only suggest relational sequences of the variables examined but not causal relationships. Empirical studies which involve increasing dairy and calcium intake should be conducted in order to better elucidate the causal relationships, if present. Second, weight and height were self-reported by participants as in-person contact during the data collection period was not allowed due to COVID-19 safety protocols. Third, the DSQ questionnaire used to quantify dairy intake in the present study did not allow for the identification of dairy sources as high-fat or low-fat, which could contribute to the lack of associations between dairy intake and some mental health measures. Additionally, the questionnaire did not provide quantification of vitamin D intake. Given that vitamin D is essential for proper calcium absorption, it is an important factor to account for when examining the effects of calcium intake. Fourth, the use of a convenience sample could limit generalizability of the results. Future studies should consider quantifying dairy intake based on fat content. Finally, while not necessarily a limitation, the study was conducted during the COVID-19 pandemic, where perceived stress, along with other negative metal health measures, were elevated among university students. The same study should be repeated after the pandemic to re-examine the relationships between dairy and calcium intake and mental health measures to confirm the results of the present study.

## 5. Conclusions

The present study noted dairy and calcium intake predicted perceived stress and positive mood scores while calcium also predicted anxiety, rumination, and resilience scores for higher education students in the U.S. Through moderation analyses, the current study also identified that, as calcium intake increased, the relationship between perceived stress and anxiety weakened as did the relationship between perceived stress and negative mood. These findings suggest that improving dairy and calcium intake, especially calcium intake, would likely result in improved mental health outcomes of higher education students. However, these conclusions need to be further examined using interventional approaches.

## Figures and Tables

**Table 1:** Demographics.

Gender n (%)	Undergraduate vs. Graduate	Domestic vs. International	Age (y) Mean ± SD	BMI (kg/m^2^) Mean ± SD	Sleep Duration (h) Mean ± SD	Sleep Quality (Scores) Mean ± SD	Dietary Risk (Scores) Mean ± SD	Physical Activity Level (METs min per Week) Mean ± SD
M = 292 (23.7)	U = 203 (69.5) G = 89 (30.5)	D = 235 (80.5) I = 57 (19.5)	24.2 ± 6.8	26.0 ± 5.7	7.5 ± 1.1	6.6 ± 3.2	8.0 ± 2.9	3730.3 ± 4386.1
F = 904 (73.3)	U = 674 (74.6) G = 230 (25.4)	D = 798 (88.3) I = 106 (11.7)	22.7 ± 5.6	25.9 ± 6.2	7.7 ± 1.3	7.6 ± 3.6	8.2 ± 2.6	3299.0 ± 4008.6
Other = 37 (3.0)	U = 27 (73.0) G = 10 (27.0)	D = 34 (91.9) I = 3 (8.1)	22.2 ± 4.0	28.6 ± 8.5	7.3 ± 1.5	9.4 ± 4.3	8.7 ± 2.3	1630.8 ± 2867.3
Total = 1233	U = 904 (73.3) G = 329 (26.7)	D = 1067 (86.5) I = 166 (13.5)	23.1 ± 5.9	26.0 ± 6.2	7.6 ± 1.3	7.4 ± 3.6	8.2 ± 2.7	3349.7 ± 4082.8

Note: M = male, F = female; other includes students who self-identified as something other than male or female, e.g., transgender, genderqueer, or other, and students who chose not to disclose. U = undergraduate, G = graduate, D = domestic, I = international, and SD = standard deviation. Sleep quality was measured using PSQI scores, with higher scores being indicative of worse sleep quality. Dietary risk was assessed using the STC questionnaire, and higher scores were consistent with higher dietary risk.

**Table 2:** Dairy and calcium intake and mental health measures.

Variables	Mean ± SD	Correlation Coefficient with Dairy Intake (*p* Value)	Correlation Coefficient with Calcium Intake (*p* Value)
Dairy intake (cups/d)	1.6 ± 0.6	-	0.920 * (*p* < 0.001)
Calcium intake (mg/d)	967.0 ± 206.0	0.920 * (*p* < 0.001)	-
Perceived stress (score)	21.6 ± 7.0	−0.062 * (*p* = 0.031)	−0.138 * (*p* < 0.001)
Anxiety (score)	9.1 ± 5.9	−0.013 (*p* = 0.662)	−0.074 * (*p* = 0.010)
Negative mood (score)	26.0 ± 8.4	<−0.001 (*p* = 0.987)	−0.053 (*p* = 0.066)
Positive mood (score)	27.0 ± 7.9	0.070 * (*p* = 0.016)	0.138 * (*p* < 0.001)
Rumination (score)	84.7 ± 23.6	0.005 (*p* = 0.864)	−0.058 ^ (*p* = 0.044)
Resilience (score)	3.2 ± 0.8	0.013 (*p* = 0.642)	0.085 * (*p* = 0.003)

Note: * indicates significant correlation. ^ indicates no longer significant after false discovery rate correction. SD = standard deviation. Perceived stress score ranges from 0 to 40, with higher scores indicating more perceived stress. Anxiety score ranges from 0 to 21, and a higher score indicates more anxiety. Negative and positive mood scores both range from 0 to 50, with higher scores indicating higher levels of negative or positive mood. Rumination scores range from 27 to 135, and higher scores represent more repetitive negative thinking. Resilience scores range from 1 to 5, with higher scores indicating more resilience.

**Table 3:** Dairy intake (cup equivalents per day) as a moderator for the relationship between perceived stress and anxiety.

Variable	B	SE	t	*p* Value
Perceived stress → anxiety	0.622	0.052	11.876	<0.001
Dairy intake → anxiety	0.848	0.637	1.332	0.183
Perceived stress × dairy intake → anxiety	−0.028	0.030	−0.920	0.358

N = 1196. Age, gender, BMI, sleep quality and duration, dietary risk, and physical activity level were adjusted for each model.

**Table 4:** Calcium intake as a moderator for the relationship between perceived stress and anxiety.

Variable	B	SE	t	*p* Value
Perceived stress → anxiety	0.724	0.086	8.418	<0.001
Calcium intake → anxiety	−0.074	0.020	−1.972	0.049
Perceived stress × calcium intake → anxiety	−0.001	0.001	−1.732	0.036
	Calcium intake (mg)	B (SE)	LL 95% CI	UL 95%CI
Conditional effects of calcium intake on the relationship between perceived stress and anxiety	806	0.604	0.558	0.649
	909	0.588	0.552	0.625
	1144	0.553	0.509	0.598

N = 1196. Age, gender, BMI, sleep quality and duration, dietary risk, and physical activity level were adjusted for each model. Calcium intakes in mg reflect 16th, 50th, and 84th percentile intakes of the sample population.

**Table 5:** Dairy intake as a moderator for the relationship between perceived stress and negative mood.

Variable	B	SE	t	*p* Value
Perceived stress → negative mood	0.904	0.075	12.048	<0.001
Dairy intake → negative mood	1.459	0.912	1.600	0.110
Perceived stress × dairy intake → negative mood	−0.043	0.043	−1.003	0.316

N = 1196. Age, gender, BMI, sleep quality and duration, dietary risk, and physical activity level were adjusted for each model.

**Table 6:** Calcium intake as a moderator for the relationship between perceived stress and negative mood.

Variable	B	SE	t	*p* Value
Perceived stress → negative mood	1.038	0.123	8.434	<0.001
Calcium intake → negative mood	−0.006	0.003	−2.248	0.025
Perceived stress × calcium intake → negative mood	−0.002	0.001	−1.666	0.046
	Calcium intake (mg)	B (SE)	LL 95% CI	UL 95%CI
Conditional effects of calcium intake on the relationship between perceived stress and negative mood	806	0.873	0.808	0.937
	909	0.851	0.799	0.904
	1144	0.803	0.740	0.867

N = 1196. Age, gender, BMI, sleep quality and duration, dietary risk, and physical activity level were adjusted for each model. Calcium intakes in mg reflect 16th, 50th, and 84th percentile intakes of the sample population.

**Table 7:** Dairy intake as a moderator for the relationship between perceived stress and positive mood.

Variable	B	SE	t	*p* Value
Perceived stress → positive mood	−0.616	0.085	−7.259	<0.001
Dairy intake → positive mood	−0.304	1.032	−0.294	0.769
Perceived stress × dairy intake → positive mood	0.040	0.049	0.826	0.409

N = 1196. Age, gender, BMI, sleep quality and duration, dietary risk, and physical activity level were adjusted for each model.

**Table 8:** Calcium intake as a moderator for the relationship between perceived stress and positive mood.

Variable	B	SE	t	*p* Value
Perceived stress → positive mood	−0.523	0.139	−3.764	<0.001
Dairy intake → positive mood	0.003	0.003	1.054	0.292
Perceived stress × dairy intake → positive mood	<0.0001	0.0001	−0.137	0.891

N = 1196. Age, gender, BMI, sleep quality and duration, dietary risk, and physical activity level were adjusted for each model.

**Table 9:** Dairy intake as a moderator for the relationship between perceived stress and rumination.

Variable	B	SE	t	*p* Value
Perceived stress → rumination	2.230	0.226	10.169	<0.001
Dairy intake → rumination	3.832	2.745	1.396	0.163
Perceived stress × dairy intake → rumination	−0.105	0.130	−0.813	0.416

N = 1196. Age, gender, BMI, sleep quality and duration, dietary risk, and physical activity level were adjusted for each model.

**Table 10:** Calcium intake as a moderator for the relationship between perceived stress and rumination.

Variable	B	SE	t	*p* Value
Perceived stress → rumination	2.424	0.371	6.534	<0.001
Dairy intake → rumination	0.009	0.008	1.194	0.233
Perceived stress × dairy intake → rumination	−0.0003	0.0004	−0.814	0.416

N = 1196. Age, gender, BMI, sleep quality and duration, dietary risk, and physical activity level were adjusted for each model.

**Table 11:** Dairy intake as a moderator for the relationship between perceived stress and resilience.

Variable	B	SE	t	*p* Value
Perceived stress → resilience	−0.064	0.008	−7.929	<0.001
Dairy intake → resilience	−0.053	0.098	−0.534	0.589
Perceived stress × dairy intake → resilience	0.001	0.005	0.277	0.782

N = 1196. Age, gender, BMI, sleep quality and duration, dietary risk, and physical activity level were adjusted for each model.

**Table 12:** Calcium intake as a moderator for the relationship between perceived stress and resilience.

Variable	B	SE	t	*p* Value
Perceived stress → resilience	−0.074	0.013	−5.620	<0.001
Dairy intake → resilience	−0.0002	0.0003	−0.811	0.417
Perceived stress × dairy intake → resilience	<0.0001	<0.0001	0.990	0.323

N = 1196. Age, gender, BMI, sleep quality and duration, dietary risk, and physical activity level were adjusted for each model.

## Data Availability

The data presented in this study are available on request from the corresponding author. The data are not publicly available due to ongoing analyses.
